# A prospective audit of paediatric cardiac MRI under general anaesthesia; practise and problems

**DOI:** 10.1186/1532-429X-11-S1-P249

**Published:** 2009-01-28

**Authors:** Marina L Hughes, Emma Stockton, Andrew Taylor, Angus McEwan

**Affiliations:** 1 Great Ormond Street Hospital for Children, London, UK; 2 Great Ormond Street Hospital, London, UK

**Keywords:** Congenital Heart Disease, Sevoflurane, Pulmonary Blood Flow, Hypoplastic Left Heart Syndrome, Cardiac Intensive Care Unit

## Background

Cardiac MRI (CMRI) is increasingly used to assist with the surgical planning and follow up of children with congenital heart disease. In small children general anaesthesia (GA) is always required. We describe our experience of GA for paediatric CMRI, using data collected prospectively over 3 years.

## Methods and results

120 patients presented for CMRI under GA from November 2005 to May 2008. These patients were aged 0 – 288 months (median 28 months), and weighed 2.8 – 64 kg (median 11.7 kg).

This cohort of patients had a large range of diagnoses, but principally comprised those with hypoplastic left heart syndrome (HLHS) and other functionally univentricular hearts for interstage imaging, patients with pulmonary atresia or Tetralogy of Fallot for surgical planning, and patients with aortic arch abnormalities.

The majority of patients (91.6%) were admitted as a day-case to our hospital on the day of CMRI. Ten patients were imaged during an inpatient stay, three of whom (2.5%) were inpatients on the Cardiac Intensive Care Unit (CICU) prior to their MRI.

Ten percent of patients were classified as American Society of Anaesthesiology (ASA) Class 2, 80% ASA Class 3 and 10% ASA Class 4.

Fifty-seven children (47.5%) had a functionally single ventricle. Fifty patients (42%) had normal oxygen saturations. Of the cyanosed patients, pulmonary blood flow was supplied through an aorto-pulmonary shunt in 33% of patients, and 13% had a cavopulmonary shunt. Sixteen patients (13%) had a mixed circulation.

Six patients (5%) had severely impaired systemic ventricular function, 10 (8.3%) had moderately impaired function and 31 (25.8%) had mildly impaired function. 14 patients (11.6%) had at least moderate systemic outflow tract obstruction.

A senior, cardiac anaesthetist was continuously present during every case. Most patients (100/120, 83%) received an inhalational induction with sevoflurane. Sixteen received an intravenous induction with propofol. The anaesthetist remained in the control room with the MRI technician and cardiologist during scanning. Breath holding was achieved from within the control room by breaking the circuit and occluding the inspiratory limb of the circle system.

The majority of children (98/120, 81.6%) had an uneventful CMRI under GA. Nineteen (15.8%) patients suffered minor adverse events. One major adverse event occurred; a patient with hypoplastic left heart syndrome (HLHS) became hypotensive and had a cardiac arrest in the MRI scanner. This patient was successfully resuscitated. One patient with HLHS died during fasting, prior to anaesthetic, on the morning of the MRI.

## Conclusion

Despite the precarious physiology of children with congenital heart disease, a GA for CMRI can be administered safely. A gas induction with sevoflurane seems safe for most children, even with impaired ventricular function. The safe provision of GA for CMRI relies on a senior multi disciplinary team working closely and regularly together. Inter-stage HLHS patients are particularly at risk of a serious adverse event and particular care should be taken to ensure adequate hydration before, during and after anaesthesia.

